# Supplementary material for: Psychosocial resources predict frequent pain differently for men and women: A prospective cohort study
Source: PLoS One. 2023 Mar 17;18(3):e0283222. doi: 10.1371/journal.pone.0283222 (PMC10022772; doi:10.1371/journal.pone.0283222)
Supplement: Attachment — Submitted filename: Response to reviewers.docx [file pone.0283222.s001.docx]

# Response to reviewers

PONE-D-22-26227

Psychosocial resources predict frequent pain differently for men and women: a prospective cohort study

**Many thanks for your valuable considerations. They were most helpful to improve the manuscript. Below, we have addressed your comments, suggestions and questions.**

| Academic editor |  |
| --- | --- |
| Please ensure that your manuscript meets PLOS ONE's style requirements, including those for file naming. | We have adjusted the manuscript according to PLOS ONE’s style requirements |
| In your Data Availability statement, you have not specified where the minimal data set underlying the results described in your manuscript can be found.  Important: If there are ethical or legal restrictions to sharing your data publicly, please explain these restrictions in detail. | The data underlying this study cannot be made freely available as they are subject to secrecy in accordance with the Swedish Public Access to Information and Secrecy Act (chapter 24, § 3 and 8).  The Health Assets Project database is stored at the Swedish National Data Service (<https://snd.gu.se/en>) under the registration number SND0870.  Requests to make data available to reproduce the findings in the study should be made to [snd@gu.se](mailto:snd@gu.se). |
| Reviewers' comments: Reviewer's Responses to Questions |  |
| 1. Is the manuscript technically sound, and do the data support the conclusions? Reviewer #1: Yes Reviewer #2: Yes |  |
| 2. Has the statistical analysis been performed appropriately and rigorously? Reviewer #1: Yes Reviewer #2: Yes |  |
| 3. Have the authors made all data underlying the findings in their manuscript fully available?  The [PLOS Data policy](http://www.plosone.org/static/policies.action#sharing) requires authors to make all data underlying the findings described in their manuscript fully available without restriction, with rare exception (please refer to the Data Availability Statement in the manuscript PDF file). The data should be provided as part of the manuscript or its supporting information, or deposited to a public repository. For example, in addition to summary statistics, the data points behind means, medians and variance measures should be available. If there are restrictions on publicly sharing data—e.g. participant privacy or use of data from a third party—those must be specified. Reviewer #1: No Reviewer #2: Yes | The data underlying this study cannot be made freely available as they are subject to secrecy in accordance with the Swedish Public Access to Information and Secrecy Act (chapter 24, § 3 and 8).  The Health Assets Project database is stored at the Swedish National Data Service (<https://snd.gu.se/en>) under the registration number SND0870.  Requests to make data available to reproduce the findings in the study should be made to [snd@gu.se](mailto:snd@gu.se). |
| 4. Is the manuscript presented in an intelligible fashion and written in standard English? Reviewer #1: Yes Reviewer #2: Yes |  |
| 5. Review Comments to the Author |  |
| In the abstract, explain what you mean by psychosocial resources upfront so that readers can understand what they can expect. | We have added “Psychosocial resources, psychological and social factors like self-efficacy and social support” in the abstract |
| Please provide the citations following proper citation style- 1-3, 4-7, not the way citations should be provided. | We have now uploaded the PLOS citation template in endnotes and used it throughout the manuscript |
| Define self-efficacy and social support in the introduction, paragraph 2, page 3, authors mention: “Generally, men have higher self-efficacy than women (8-10), and women give and receive more social support than men (7, 11, 12).” Also, define what you mean by instrumental social support upfront so that readers can follow smoothly. | Thank you for your important consideration. We agree that definitions were missing and have added definitions for general self-efficacy, social support, emotional and instrumental social support |
| Explain the Swedish version, validated in 2012 measurement, very clearly so readers can understand better. | We have rephrased the sentence |
| In this paragraph containing “The scores of two of the questions (“Do you have a relative or a friend who is willing to help you if you are sick?”: I think it would be better not to use the word “scores” as it confuses readers with what “scores” the authors are talking about. Instead, authors can say “answers” and later explain how the answers are combined to get a 0-1 score. | Thank you for your suggestion. We agree that it makes the text more comprehensible. We have changed “scores” to “answers” |
| Explain what the categories are. - Age (3 categories), education (3 categories), and country of birth (2 categories) were used as control variables. | We have added information about the categories |
| It will be easier to understand the dependent and the independent variables if the authors present an equation. I understood frequent pain = f(baseline score of psychosocial resources by sex, control). Is frequent pain a binary measurement? How are all the questions for measuring dependent variables combined? | Yes, frequent pain is a binary variable. We agree that there is a need of clarification and have added information about how the questions for measuring the dependent variables are combined |
| Are these psychosocial resources run separately during the estimation? I thought the model would likely be frequent pain = f(men and women GSE, ISS, ESS, control). The separating solid black lines in Table 2 between GSE, ISS, and ESS are confusing. | The analyses for GSE, ISS and ESS were run separately. As we conducted three different estimations, those were presented as three different parts in Table 2 |
| Overall comments: check the citation/grammar. | We have checked citations and grammar carefully to ensure correct language |
| Reviewer 2 |  |
| The article is interesting i would suggest to, if possible add the time ( duration ) should be taken into account to something like pain. The subject is current and the way you organized the article is quite well structured. | We agree that duration is an important aspect in pain. The time aspects available in this study were questions about pain frequency during the past twelve month. We agree, in future studies, pain duration and the associations between pain duration, self-efficacy and social support should be explored in more detail |
| 6. PLOS authors have the option to publish the peer review history of their article ([what does this mean?](https://journals.plos.org/plosone/s/editorial-and-peer-review-process#loc-peer-review-history)). If published, this will include your full peer review and any attached files. If you choose “no”, your identity will remain anonymous, but your review may still be made public. | Thank you for this information |
